# Large-Scale Age-Dependent Skewed Sex Ratio in a Sexually Dimorphic Avian Scavenger

**DOI:** 10.1371/journal.pone.0046347

**Published:** 2012-09-27

**Authors:** Sergio A. Lambertucci, Martina Carrete, José Antonio Donázar, Fernando Hiraldo

**Affiliations:** 1 Laboratorio Ecotono, INIBIOMA (CONICET-Universidad Nacional del Comahue), Bariloche, Argentina; 2 Department of Physical, Chemical and Natural Systems, University Pablo de Olavide, Sevilla, España; 3 Departmento de Biología de la Conservación, Estación Biológica de Doñana, CSIC, Sevilla, España; National Cancer Institute, United States of America

## Abstract

Age-dependent skewed sex ratios have been observed in bird populations, with adult males generally outnumbering females. This trend is mainly driven by higher female mortality, sometimes associated with anthropogenic factors. Despite the large amount of work on bird sex ratios, research examining the spatial stability of adult sex ratios is extremely scarce. The Andean condor (*Vultur gryphus*) is the only bird of prey with strong sexual dimorphism favouring males (males are 30% heavier than females). By examining data from most of its South-American range, we show that while the juvenile sex ratio is balanced, or even female-skewed, the sex ratio becomes increasing male-skewed with age, with adult males outnumbering females by >20%, and, in some cases by four times more. This result is consistent across regions and independent of the nature of field data. Reasons for this are unknown but it can be hypothesized that the progressive disappearance of females may be associated with mortality caused by anthropogenic factors. This idea is supported by the asymmetric habitat use by the two sexes, with females scavenging in more humanized areas. Whatever the cause, male-skewed adult sex ratios imply that populations of this endangered scavenger face higher risks of extinction than previously believed.

## Introduction

Literature on the theory of sex ratios is abundant but the structure and sex ratio of animal populations is less well known [1]. Reports showing skewed sex ratios with age are available for a number of vertebrate species, mostly birds [2], [3]. Male-skewed adult sex ratios (ASR) are the rule, being probably driven by higher rates of female mortality [2]. A skewed sex ratio favouring males can produce an increase in sexual aggression towards females, and hence a further reduction in their survival (and increase in skew in the sex ratio) [4], [5]. Male-skewed ASR is more pronounced in wild populations of threatened species which could, in some cases, be explained by increasing intersexual competition for scarce resources [2]. Threats associated with human-induced environmental alterations may cause or accelerate these trends [6]–[8]. Finally, distortions in the ASR may result in a reduction in population viability [2].

Most of literature on ASR in birds concerns single-populations [2]. Large-scale approaches describing how ASR varies for a single species throughout its distribution range are lacking, with the exception of some small passerines in temperate regions [2]. Such studies however, can provide key insights into both ecological and conservation issues. For instance, large-scale studies allow spatial differences in ASR to be related to intrinsic (population) and extrinsic (environmental) characteristics and can help to resolve the adaptive value of changes in the proportion of sexes. Sex-ratio skew could be used as a proxy tool for identifying declining populations, and may alert conservationists to declines that were undetected by census data focused on abundances of individuals [9]. Then, if a skewed ASR is found throughout the whole species’ range, this may be cause for increased conservation concern.

**Figure 1:**
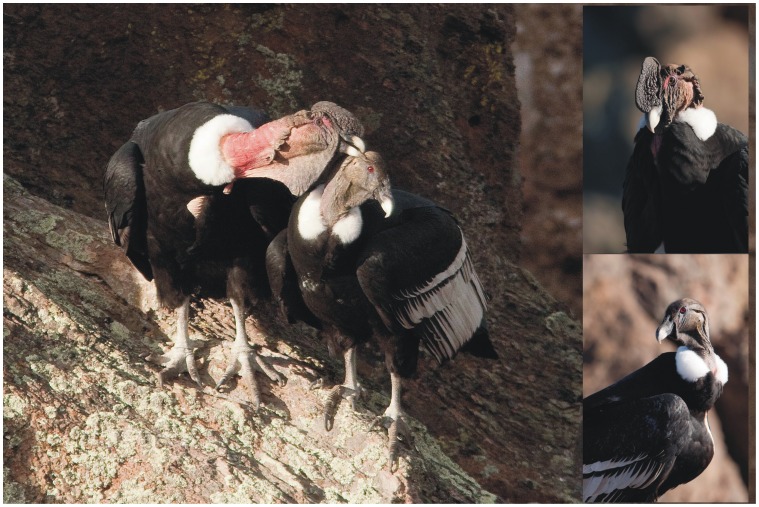
Phenotypic differences among Andean condor sexes. Males (left and above) are larger than females, have a comb and a brown iris; females (right and below) have a red iris and no comb (Photos: M. Diéz Peña).

Here, we examine changes in sex ratio in relation to age for the largest bird scavenger in the world, the Andean condor (*Vultur gryphus*), throughout its South-American range. Males weigh 30% more than females (ca. 15 kg vs. 11 kg respectively; authors unpublished data), making this species the unique exception to the widespread reversed sexual dimorphism found in birds of prey (Figure 1) [10], [11]. The Andean condor shows extreme life-history patterns (high longevity and slow reproductive rates [10]), making it very vulnerable to human-induced threats [12]–[14]. In fact, the species is considered as “Near Threatened” and is included in CITES I due to the generalized declines observed in many parts of its range [12]. We use field data and a complete literature survey to describe how sex ratio varied in relation to age (from young to adult) within the main distribution of the species in South America. For this purpose we performed two analyses on the sex ratio of condors: 1) a temporal survey of communal roosts; and 2) spatial surveys of condors feeding, foraging and roosting in different areas of their range.

## Methods

### Study Species

The Andean condor lives in hilly areas throughout the South American Andes region. They are social birds aggregating at carcasses and roosting sites. Large cliffs are used as communal roosts where birds of different sex and age may segregate, with adult males occupying the sunniest and sheltered shelves, but with all classes present in the roost [15], [16]. This is a highly despotic species with adult males being at the top of the hierarchy and juvenile females at the lower rank [11]. In general, aggression is directed at those individuals belonging to lower hierarchical levels, a behaviour that can produce despotic habitat use [11]. Consequently, males (dominants) and females (subordinates) are generally segregated in relation to foraging areas using different microhabitat structures. Males select more rugged higher-quality areas whereas females are more frequently detected in more humanized plains (valleys) [11], [13].

### Temporal Trends on Sex Ratio

Firstly, data were collected from a large communal roost in Northwest of Patagonia (near Bariloche city), southern Argentina (40° 50′S; 71° 2′W), where we performed one census per day (a “time series”), during January 2006. Permissions to monitor condors were provided by Dirección de Fauna Silvestre de Río Negro, the Argentine National Park Administration, and the owners and managers of local farms. In addition, we used published “time series” data collected from a large communal roost in central Argentina (Córdoba [17]). In both cases methods were similar, following standardized procedures [16]–[18]. Censuses were performed by two to three observers visiting each roost daily to count condors from blinds with the aid of telescopes and binoculars. One census per day was considered, but birds were counted twice daily: at dusk, when individual condors could still be observed and most were roosting rather than flying and at first light the next morning, before condors left roost sites. For each day, the total number of birds roosting was recorded, distinguishing between two age classes (adults and immatures), or three when reported (juveniles, subadults and adults) on the basis of plumage patterns, as well as sex by the presence or absence of a comb (Figure 1) [10], [19]. Each time series for an individual roost was analysed by means of Randomization tests [20], on the basis of the trends shown by each pair (number males vs. females in each count). Our null hypothesis was that differences in sex ratio were due to chance [20]. To provide information on the variability in the temporal use of communal roosts, we also included the percentage of times males were more abundant than females and the coefficient of variation (CV) in the male to female ratio for an entire set of censuses.

### Spatial Patterns on Sex Ratio

**Figure 2:**
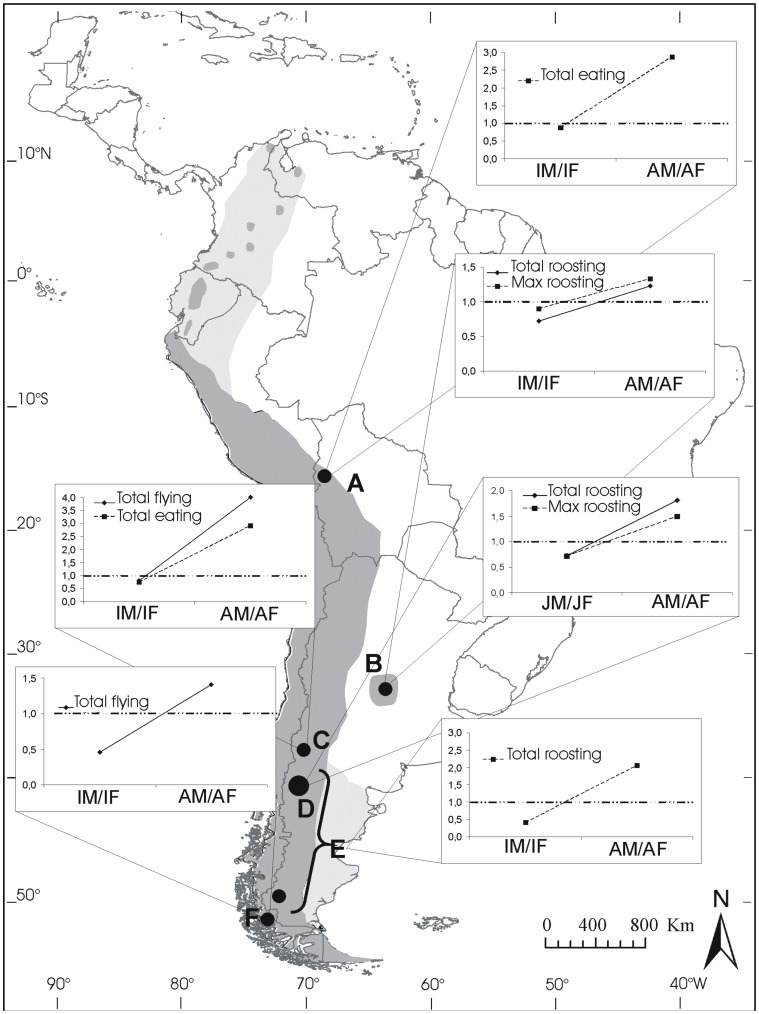
Andean condor distribution and sex ratios. Distribution of Andean condors (historic: light grey, and current: dark grey) showing the localities (black points) of studies reporting sex-age classes. Inset graphs indicate sex ratios (male:female) for each age class, the dotted line indicates a balanced sex ratio. Data correspond to the maximum (Max) and the total (Total) number of individuals reported in each survey (see methods for more detail on definitions). Estimates of sex ratio were made by means of: direct observations in communal roosts (B; D), molecular sexing of moulted feathers collected in roosts (E), observations of birds performing foraging flights (C; F), or feeding at carcases (A; C). Localities correspond to: A: Apolobamba Mountains, Bolivia; B: Córdoba, Argentina; C: Neuquén, Argentina; D: Northwest of Patagonia, Argentina; E: Patagonia, Argentina; and F: Torres del Paine, Chile.

The second analysis was performed using own and published data on the Andean condor sex ratio in different localities of South America covering the main species’ range, from northern Bolivia to southern Patagonia, Argentina and Chile (more than 4,000 km; Fig. 2). These studies describe the age and sex of individuals seen at carcasses, flying, or roosting. For each particular study we determined: a) the total number of birds counted during the entire survey (i.e., the sum of all observed birds); and/or b) the maximum number of birds, which was defined as the highest number of individuals recorded at any time. In each case, the statistical significance of departure of the observed sex ratios from equality (0.5) was assessed by binomial tests [21].

The information on Andean condor sex ratio from the literature was obtained with different methods and in various locations (Table 1). Feijóo [17] estimated the abundance of individuals by monitoring the largest communal roost located in the centre of the distribution of the species in Córdoba, Argentina. These censuses were conducted throughout the four seasons during two years. Rios-Uzeda and Wallace [22] estimated the maximum number of individuals of each age and sex at 6 carcasses located in northwest Bolivia (within an area ranging from ca. 3700 to 5000 m asl, and more than 70 km north-south). Donázar et al. [11] assessed the abundance of individuals of each age and sex flying (foraging birds) and feeding (birds at carcasses) in both mountain and plain areas in different seasons in Neuquén Province, north of Patagonia. Sarno et al. [23] counted birds flying (foraging birds) in different areas in Torres del Paine National Park, Chile. Although they provide data on the abundance of individuals of each age and sex in mountains and valleys, they pooled the data into a single dataset without discriminating by habitat type. Finally, Alcaide et al. [24] estimated the minimum number of individuals of each age and sex class in Patagonia by molecular sexing and genetic analyses of feathers collected at 15 communal roosts located throughout Patagonia (Neuquén, Río Negro, and Santa Cruz provinces), Argentina. The accuracy of molecular sexing was tested by means of repeated analyses of a control individual.

**Table 1:** Sex ratio (male to female) of Andean condors in different regions within the distribution range of the species.

Location	Year	Survey^1^	Type of	Adult					Immature				
			data	Male	Female	%Male	P	Male	Female	%Male	P	N	Source
Córdoba,Argentina	1997–1998	Roosting birds	Maximum	32	24	0.57	ns	26	29	0.47	ns	35 censuses	[17]
			Total	645	526	0.55	**	557	776	0.42	**	2504 individuals	[17]
Apolobamba Mountains,Bolivia	2005	Birds at carcasses	Maximum	23	8	0.74	*	22(7J+15SA)	25(17J+8SA)	0.47	ns	78 individuals	[22]
Neuquén, Argentina	1991–1992, 1995	Foraging birds	Total	36	9	0.80	**	32	40	0.44	ns	117 individuals	[11]
		Birds at Carcasses	Total	105	36	0.74	**	50	68	0.42	ns	259 individuals	[11]
Torres del Paine, Chile	1992–1994	Foraging birds	Total	267	190	0.58	**	91	200	0.31	**	748 individuals	[23]
Patagonia, Argentina	2007	Roosting birds	Maximum	33	16	0.67	*	9	22	0.29	*	80 individuals	[24]
Northwest of Patagonia, Argentina	2006	Roosting birds	Maximum	18	12	0.60	ns	11(5J+6SA)	10(7J+3SA)	0.52	ns	19 censuses	[This study]
			Total	172	95	0.64	**	51(27J+24SA)	50(37J+13SA)	0.50	ns	368 individuals	[This study]

Data on sexes is separated by age-class (adult vs. immature, the latter including juveniles and subadults). Surveys included in this table were made by field observations of birds roosting in communal roosts [17] (and this study), performing foraging flights [23], or feeding at carcasses [11] and [22], and molecular sexing of moulted feathers collected at communal roosts [24]. We included data on the maximum number of individuals (Maximum) and the total number of individuals counted in a survey (Total; see methods for more details).

1 Condor behaviour at the time of survey (i.e. foraging, feeding, or roosting).

AM/AF = Adult male to adult female; IM/IF = Immature male to immature female; JM/JF = Juvenile male to juvenile female; J = Juvenile; SA = Subadult;

P = significance; ns = no significant, *≤0.05, **≤0.001 (Binomial test).

## Results

### Temporal Trends

When examining time series of daily counts in roosting areas of Patagonia (southern Argentina) we found that sex ratios of immature birds did not differ from 0.5 (Randomization test, Randomizations = 1000; N = 19; P = 0.9). In contrast, adult male outnumbered significantly adult females (Randomization test, Randomizations = 1000; N = 19; P = 0.01). There were more adult males than females on 100% of census days, and more immature males than females on 44.4% of census days. The coefficient of variation (CV) for adult and immature sex ratios was 0.63 and 0.69, respectively.

Daily counts conducted in roosts in Córdoba (central Argentina) showed that immature females were more numerous than males (Randomization test, Randomizations = 1000; N = 35; P<0.01); and, again more adult males were observed than females (Randomization test, Randomizations = 1000; N = 35; P = 0.035). There were more adult males than females on 87.5% of the days, and more immature males than females on 11.4% of the days. The CV for adult and immature sex ratios was 0.70 and 0.30, respectively.

### Spatial Patterns

Our second analysis showed that increasing male-skewed sex ratios with age was the pattern throughout the main species range in South America. Adult males were always 20% more numerous than females, reaching up to four times more, with a mean value of ca. 162% (Table 1). Juvenile sex ratio was always balanced or even slightly skewed towards females, whereas adult sex ratio was invariably skewed to males (Figure 2). This trend was common to data for flying, roosting and feeding birds (Table 1). Although the sample size for the estimation of the maximum number of individuals at the roosting sites can be low, such that the sex ratio differences are either not significant, or less significant than ratio data collected from other sources, all the tendencies were consistent (see Table 1 and Figure 2). Importantly the molecular sexing of feathers from different individuals collected at roosts agrees with the observations made at the roosts (Figure 1).

Articles reviewed surveyed condors from different habitats (including plains and hilly areas), however, most do not separate the results by habitats. An exception is Donázar et al. [11], who found that the adult male to female ratio in mountains and plains favours males in both foraging and feeding birds (proportion of males *flying*: in mountains 0.81 adults and 0.40 immatures versus 0.79 adults and 0.31 immatures in plains; proportion of males *eating*: in mountains 0.78 adults and 0.42 immatures versus 0.60 adults and 0.42 immatures in plains).

On the other hand, the sex ratios of immature birds separating them into subadults and juveniles were recorded in only two studies. In those cases, subadult sex ratios approached that of adults (proportion of males for Bolivia: 0.74 adults, 0.65 subadults and 0.29 juveniles; and for Patagonia: 0.60 adults, 0.67 subadults and 0.42 juveniles; Table 1) supporting the idea that ASR progressively skewed with age.

## Discussion

In this paper we show a consistent male-skewed adult sex ratio in wild populations of a threatened species, the Andean condor, throughout most of its geographical distribution, in South America. In contrast, the juvenile sex ratio was balanced or even female-skewed, suggesting that sex ratio at fledging is probably near 0.5. Our results are based on data from different articles and may have some biases regarding the specific features of the sites selected for sampling in each study. However, the stability of the pattern found across data types (i.e., carcasses located in different areas such as mountains and plains, censuses and collection of feathers from different communal roosts, and censuses of individuals flying in different areas) allows us to be confident of the validity of the results and call for an urgent consideration of the problem

The estimation of the sex ratios in the wild by means of direct observation has been shown to be very reliable [2]. We found low variability in the proportion of adult males and females in the communal roosts sampled throughout days, and adult males outnumbered females most, if not all, of the time. Moreover, the genetic analysis of feathers collected at roosting places in our study area also showed a male-skewed sex ratio in adult birds [24], supporting the results from direct observations on communal roosts. Although male condors outcompete females when feeding, which may lead to a despotic spatial distribution, this does not seem to introduce a false representation of the sexes since in both areas, mountains and plains, the skew in the sex ratio has the same tendency (i.e., males always outnumbered females) [11]. Finally, it is unlikely that our results were influenced by asymmetric parental roles, as, though information is still scarce, males seem to spend a similar amount of time in the nesting area as females or more [19], [25]

Skewed sex ratios are found in a wide range of animal groups including insects, fish, reptiles, mammals, and birds [2], [26]–[28] (but see [29]). The causes for such biases can be evolutionary, ecological, environmental or anthropic [30]. The main driver of male-skewed ASR for many animals, particularly birds, is a higher rate of mortality in the less common sex, as opposed to the sex ratio of offspring [2]. We suggest that this may also be the main factor explaining our results as sex ratio of juveniles is balanced (or slightly skewed toward females) but that of subadults and adults is male-skewed. Although the aim of this paper was to describe the pattern and not to test among alternative hypotheses explaining skewed ASR, we suggest that our results could arise as a consequence of sexual habitat segregation, since female Andean condors tend to forage preferentially in plains where risks associated with human activities are higher [11], [13], [31]. Anthropogenic disturbances may have a disproportionate effect on juvenile birds (mainly for females), which are much more confident toward humans [32]. Thus, an evolutionary strategy leading to sexual segregation by habitat may now result in a maladaptive trait as risks associated to human activities are higher in habitats more frequently used by one sex, a situation also found in other vertebrates [7], [8]. However, because of the nature of our study, we cannot rule out other factors that can produce female-skewed mortality. For example, males have been known to kill females in captivity [S. Feo pers. comm.]. Furthermore, body size and lifespan are always related [33], suggesting that males could have longer life expectancy than females. Further research requiring detailed monitoring of condor populations is needed to disentangle plausible hypotheses. Nonetheless, intersexual and interspecific competition for scarce resources, coupled with human threats, could be influencing sex ratios and merit special attention in this sexually-despotic species [11], [13]

Andean condor populations have been reduced throughout most of South America, being nearly extinct in the northern parts of their range [12]. Larger numbers are found in the southern part of the continent (Patagonia) but an important proportion of these individuals are non-reproductive birds [16], [18]. From a conservation point of view, it is clear that a skewed sex ratio, whatever the cause that promotes it, reduces the effective population size, increasing the extinction risk [2]. Special attention should be paid to the role that intersexual behavioural asymmetries could play in the observed skewed sex ratios, as human-induced factors can exacerbate this phenomenon. In some animal species, adult biases in the sex ratio may strongly influence the behaviour of individuals, making males more aggressive for example, and thereby increasing the competition with (and the negative effects on) females, which can in turn impact population dynamics [4], [5]. If human-induced mortality is affecting females disproportionately, Andean condors might have fallen into an “evolutionary trap”, which could lead to an even more drastic reduction in population viability [5], [34]. Finally, we wish to highlight the relevance of these themes to reintroduction programs. Andean condors from captive breeding and rehabilitation centres are being reintroduced in many areas of South America [12]. Most reports on these projects do not reveal information on sex ratio of the released individuals, but the scarce data available suggest that males may be outnumbering females (e.g. 36 males and 29 females released in Colombia between 1989–2005, [35]). Juvenile sex ratios that mimic the patterns observed in the wild would be desirable in order to avoid adding further biases in the populations, which would contribute to the decrease of their long-term viability

## References

[1] Orzack SH (2002) Using sex ratios: the past and the future. Sex ratios: concepts and research methods Cambridge University Press, Cambridge: 383–398.

[2] Donald PF (2007) Adult sex ratios in wild bird populations. Ibis 149: 671–692.

[3] Ruckstuhl KE, Neuhaus P (2005) Sexual segregation in vertebrates: ecology of the two sexes. Cambridge Univ Press. 488 p.

[4] Kvarnemo C, Ahnesjo I (1996) The dynamics of operational sex ratios and competition for mates. Trends in Ecology & Evolution 11: 404–408. 21237898 10.1016/0169-5347(96)10056-2

[5] Le Galliard J-F, Fitze PS, Ferrière R, Clobert J (2005) Sex ratio bias, male aggression, and population collapse in lizards. Proceedings of the National Academy of Sciences of the United States of America 102: 18231. 16322105 10.1073/pnas.0505172102 PMC1312374

[6] Ferrer M, Hiraldo F (1992) Man-induced sex-biased mortality in the Spanish imperial eagle. Biological Conservation 60: 57–60.

[7] Darden SK, Croft DP (2008) Male harassment drives females to alter habitat use and leads to segregation of the sexes. Biology letters 4: 449–451. 18682356 10.1098/rsbl.2008.0308 PMC2610095

[8] Mucientes GR, Queiroz N, Sousa LL, Tarroso P, Sims DW (2009) Sexual segregation of pelagic sharks and the potential threat from fisheries. Biology Letters 5: 156–159. 19324655 10.1098/rsbl.2008.0761 PMC2665836

[9] Brooke M de L, Flower TP, Campbell EM, Mainwaring MC, Davies S, et al (2012) Rainfall-related population growth and adult sex ratio change in the Critically Endangered Raso lark (Alauda razae). Animal Conservation, in press. doi 0 (1111/j.1469-1795.2012.00535): x.

[10] del Hoyo J, Elliott A, Sargatal J, editors (1994) Handbook of the birds of the world vol II: New world vultures to guinea fowl. Lynx Edicions. Barcelona. 638 p.

[11] Donázar JA, Travaini A, Ceballos O, Rodríguez A, Delibes M, et al (1999) Effects of sex-associated competitive asymmetries on foraging group structure and despotic distribution in Andean condors. Behavioral Ecology and Sociobiology 45: 55–65.

[12] BirdLife International (2012) Species factsheet: Vultur gryphus. http://www.birdlife.org (accessed 10-Jul-2012).

[13] Carrete M, Lambertucci SA, Speziale K, Ceballos O, Travaini A, et al (2010) Winners and losers in human-made habitats: interspecific competition outcomes in two Neotropical vultures. Animal Conservation 13: 390–398.

[14] Lambertucci SA, Donázar JA, Huertas AD, Jiménez B, Sáez M, et al (2011) Widening the problem of lead poisoning to a South-American top scavenger: Lead concentrations in feathers of wild Andean condors. Biological Conservation 144: 1464–1471.

[15] Donázar JA, Feijóo JE (2002) Social structure of Andean Condor roosts: influence of sex, age, and season. The Condor 104: 832–837.

[16] Lambertucci SA (2010) Size and spatio-temporal variations of the Andean condor Vultur gryphus population in north-west Patagonia, Argentina: communal roosts and conservation. Oryx 44: 441–447.

[17] Feijóo J (1999) Primer censo de cóndores para la Quebrada del Condorito. Registro Nacional del Cóndor Andino, Zoológico de Buenos Aires 7: 18–25.

[18] Lambertucci SA, Luis Jácome N, Trejo A (2008) Use of communal roosts by Andean Condors in northwest Patagonia, Argentina. Journal of Field Ornithology 79: 138–146.

[19] McGahan J (1972) Behaviour and ecology of the Andean Condor [PhD Thesis]. Madison, USA: University of Wisconsin.

[20] Manly BF. (2007) Randomization, bootstrap and Monte Carlo methods in biology. Chapman & Hall/CRC. 456 p.

[21] Wilson K, Hardy IC (2002) Statistical analysis of sex ratios: an introduction. In: Hardy IC, editor. Sex Ratios: Concepts and Research Methods. 48–92.

[22] Ríos-Uzeda B, Wallace RB (2007) Estimating the size of the Andean Condor population in the Apolobamba Mountains of Bolivia. Journal of Field Ornithology 78: 170–175.

[23] Sarno RJ, Franklin WL, Prexl WS (2000) Actividad y características poblacionales de los Cóndores Andinos en el sur de Chile. Revista Chilena de Historia Natural 73: 3–8.

[24] Alcaide M, Cadahía L, Lambertucci SA, Negro JJ (2010) Noninvasive estimation of minimum population sizes and variability of the major histocompatibility complex in the Andean condor. The Condor 112: 470–478.

[25] Lambertucci SA, Mastrantuoni OA (2008) Breeding behavior of a pair of free-living Andean Condors. Journal of Field Ornithology 79: 147–151.

[26] Stoks R (2001) What causes male-biased sex ratios in mature damselfly populations? Ecological Entomology 26: 188–197.

[27] Aresco MJ (2005) The effect of sex-specific terrestrial movements and roads on the sex ratio of freshwater turtles. Biological Conservation 123: 37–44.

[28] Christe P, Keller L, Roulin A (2006) The predation cost of being a male: implications for sex-specific rates of ageing. Oikos 114: 381–384.

[29] Wehi PM, Nakagawa S, Trewick SA, Morgan-Richards M (2011) Does predation result in adult sex ratio skew in a sexually dimorphic insect genus? Journal of Evolutionary Biology 24: 2321–2328. 21848984 10.1111/j.1420-9101.2011.02366.x

[30] Hardy ICW (2002) Sex Ratios: Concepts and Research Methods. Cambridge University Press. 442 p.

[31] Speziale KL, Lambertucci SA, Olsson O (2008) Disturbance from roads negatively affects Andean condor habitat use. Biological Conservation 141: 1765–1772.

[32] Lambertucci SA, Speziale KL (2009) Some possible anthropogenic threats to breeding Andean condors (Vultur gryphus). Journal of Raptor Research 43: 245–249.

[33] Speakman JR (2005) Body size, energy metabolism and lifespan. Journal of Experimental Biology 208: 1717–1730. 15855403 10.1242/jeb.01556

[34] Schlaepfer MA, Runge MC, Sherman PW (2002) Ecological and evolutionary traps. Trends in Ecology & Evolution 17: 474–480.

[35] Ministerio de Ambiente, Vivienda y Desarrollo Territorial (2006) Programa Nacional para la Conservación del Cóndor Andino en Colombia: Plan de acción 2006–2016. Colombia: Jotamar Ltda. 32 p.

